# Supplementary material for: Dynamic epistasis analysis reveals how chromatin remodeling regulates transcriptional bursting
Source: Nat Struct Mol Biol. 2023 May 1;30(5):692–702. doi: 10.1038/s41594-023-00981-1 (PMC10191856; doi:10.1038/s41594-023-00981-1)
Supplement: Supplementary file 1 — Reporting Summary [file 41594_2023_981_MOESM1_ESM.pdf]

Corresponding author(s): Tineke Lenstra

Last updated by author(s): Dec 28, 2021

Reporting Summary

Nature Portfolio wishes to improve the reproducibility of the work that we publish. This form provides structure for consistency and transparency in reporting. For further information on Nature Portfolio policies, see our Editorial Policies and the Editorial Policy Checklist.

Statistics

For all statistical analyses, confirm that the following items are present in the figure legend, table legend, main text, or Methods section.

n/a	Confirmed	
☐	☒	The exact sample size (*n*) for each experimental group/condition, given as a discrete number and unit of measurement
☐	☒	A statement on whether measurements were taken from distinct samples or whether the same sample was measured repeatedly
☐	☒	The statistical test(s) used AND whether they are one- or two-sided<br>*Only common tests should be described solely by name; describe more complex techniques in the Methods section.*
☒	☐	A description of all covariates tested
☐	☒	A description of any assumptions or corrections, such as tests of normality and adjustment for multiple comparisons
☐	☒	A full description of the statistical parameters including central tendency (e.g. means) or other basic estimates (e.g. regression coefficient) AND variation (e.g. standard deviation) or associated estimates of uncertainty (e.g. confidence intervals)
☒	☐	For null hypothesis testing, the test statistic (e.g. F, t, r) with confidence intervals, effect sizes, degrees of freedom and P value noted<br>*Give P values as exact values whenever suitable.*
☒	☐	For Bayesian analysis, information on the choice of priors and Markov chain Monte Carlo settings
☒	☐	For hierarchical and complex designs, identification of the appropriate level for tests and full reporting of outcomes
☒	☐	Estimates of effect sizes (e.g. Cohen's d, Pearson's r), indicating how they were calculated

Our web collection on statistics for biologists contains articles on many of the points above.

Software and code

Policy information about availability of computer code

Data collection	Micromanager software (version 1.4) for acquisition of fluorescence imaging
Data analysis	Software code for analysis of transcription dynamics microscopy data is available at https://github.com/Lenstralab/livecell. Software code for analysis of smFISH microscopy data is available at https://github.com/Lenstralab/smFISH. Software code for analysis of MNase-seq data is available at https://github.com/Lenstralab/MNase_analysis.

For manuscripts utilizing custom algorithms or software that are central to the research but not yet described in published literature, software must be made available to editors and reviewers. We strongly encourage code deposition in a community repository (e.g. GitHub). See the Nature Portfolio guidelines for submitting code & software for further information.

Data

Policy information about availability of data

All manuscripts must include a data availability statement. This statement should provide the following information, where applicable:

- Accession codes, unique identifiers, or web links for publicly available datasets
- A description of any restrictions on data availability
- For clinical datasets or third party data, please ensure that the statement adheres to our policy

The MNase-seq datasets generated during the current study are available in the NCBI's Gene Expression Omnibus61 through GEO Series accession number GSE190737 (https://www.ncbi.nlm.nih.gov/geo/query/acc.cgi?acc=GSE190737). For MNase-seq, reads were aligned to the reference genome SacCer3 (January 2015). MNase-seq metagene plots were generated using all verified open reading frames in the Saccharomyces Genome Database (SGD) (Cherry, J. M. et al. Saccharomyces Genome Database: The genomics resource of budding yeast. Nucleic Acids Res. 40, 700–705 (2012).)). TATA and TATA-mismatch genes were identified as 'TATA-containing' and 'TATA-less' as previously described (Rhee, H. S. & Pugh, B. F. Genome-wide structure and organization of eukaryotic pre-

initiation complexes. Nature 483, 295–301 (2012).). Source data are provided with this paper. The microscopy data generated during the current study are available from the corresponding author on reasonable request.

Field-specific reporting

Please select the one below that is the best fit for your research. If you are not sure, read the appropriate sections before making your selection.

☒ Life sciences ☐ Behavioural & social sciences ☐ Ecological, evolutionary & environmental sciences

For a reference copy of the document with all sections, see nature.com/documents/nr-reporting-summary-flat.pdf

Life sciences study design

All studies must disclose on these points even when the disclosure is negative.

Sample size	For live-cell fluorescence imaging, at least 100 cells were included for each condition, from at least 3 biological replicates imaged in independent experiments. This number of cells allows to robustly determine transcriptional bursting parameters, while the use of independent biological replicates eliminated bias that may have occurred because of unwanted additional random mutations in the yeast strains. For MNase-seq, two replicate experiments were performed for most conditions, as indicated in the figure legends.
Data exclusions	One dataset was excluded from the MNase-seq analysis based on the digestion levels as assessed on agarose gel.<br>For live-cell imaging, cells that were segmented incorrectly and cells that contained tracking errors were excluded from analysis. In addition, we carefully checked the distributions of transcriptional bursting parameters in each biological replicate indivudally. We then found two samples (the -Taf1 dataset described in Fig. 6 and Extended Data Fig. 8 and the +Mot1&RSC dataset described in Fig. 5 and Extended Data Fig. 8) contained a fast-inducing subpopulation that arose from a single replicate experiment, suggesting that the subpopulation arose from technical rather than biological variation. The deviations of these samples may be caused by off-target mutations in these replicates, or from experimental error (for example by accidentally pre-growing cells in media with galactose instead of raffinose). When we checked how these replicates affected the analysis, we observed that exclusion of these replicates from the data resulted in the same synergies and conclusions from our dynamic epistasis analysis. Although we generally do not cherry-pick or remove outliers, we felt that the best approach is to remove these individual replicate experiments from the datasets, since the results are the same in both cases and this prevents potential overinterpretation of the subpopulations by readers.
Replication	all replication attempts were succesful, except the 2 experiments mentioned in the previous point.
Randomization	Randomization is not relevant to this study, as data was not subdivided into different experimental groups.
Blinding	Blinding is not relevant to this study, as data was not subdivided into different experimental groups.

Reporting for specific materials, systems and methods

We require information from authors about some types of materials, experimental systems and methods used in many studies. Here, indicate whether each material, system or method listed is relevant to your study. If you are not sure if a list item applies to your research, read the appropriate section before selecting a response.

Materials & experimental systems

n/a	Involved in the study	
☐	☒	Antibodies
☒	☐	Eukaryotic cell lines
☒	☐	Palaeontology and archaeology
☒	☐	Animals and other organisms
☒	☐	Human research participants
☒	☐	Clinical data
☒	☐	Dual use research of concern

Methods

n/a	Involved in the study	
☒	☐	ChIP-seq
☒	☐	Flow cytometry
☒	☐	MRI-based neuroimaging

Antibodies

Antibodies used	Western blot analysis was performed using antibodies against V5 (ThermoFisher R960-25, RRID: AB_2556564), Pgk1 (Invitrogen 22C5D8, RRID: AB_2532235), histone H3 (RRID:AB_2631108, a kind gift of the F.v.L. laboratory)(Frederiks, F. et al. Nonprocessive methylation by Dot1 leads to functional redundancy of histone H3K79 methylation states. Nat. Struct. Mol. Biol. 15, 550–557 (2008).) and histone H3K79me3 (RRID:AB_2631107, a kind gift of the F.v.L. laboratory)(Frederiks, F. et al. Nonprocessive methylation by Dot1 leads to functional redundancy of histone H3K79 methylation states. Nat. Struct. Mol. Biol. 15, 550–557 (2008).). Secondary antibodies used were IRDye 800CW Goat anti-Mouse IgG 925-32210 Li-COR (RRID AB_2687825), IRDye 800CW Goat anti-Rabbit IgG 926-32211 Li-COR (RRID:AB_621843) and IRDye 680RD Donkey anti-Mouse IgG 925-68072 Li-COR (RRID AB_2814912).
Validation	Validation information can be found at the folllowing websites for the following proteins:<br>V5 (https://www.thermofisher.com/antibody/product/V5-Tag-Antibody-Monoclonal/R960-25)

Pgk1 (https://www.thermofisher.com/antibody/product/PGK1-Antibody-clone-22C5D8-Monoclonal/459250)
Goat anti-Mouse (https://www.licor.com/bio/reagents/irdye-800cw-goat-anti-mouse-igg-secondary-antibody)
Goat anti-Rabbit (https://www.licor.com/bio/reagents/irdye-800cw-goat-anti-rabbit-igg-secondary-antibody)
Donkey anti-Mouse (https://www.licor.com/bio/reagents/irdye-680rd-donkey-anti-mouse-igg-secondary-antibody)

The following antibodies were kind gifts from the Fred van Leeuwen laboratory (The Netherlands Cancer Institute). Both antibodies were validated in (Frederiks, F. et al. Nonprocessive methylation by Dot1 leads to functional redundancy of histone H3K79 methylation states. Nat. Struct. Mol. Biol. 15, 550–557 (2008).):
histone H3
histione H3K79me3
