# Supplementary figures and images for: Dynamic epistasis analysis reveals how chromatin remodeling regulates transcriptional bursting
Source: Nat Struct Mol Biol. 2023 May 1;30(5):692–702. doi: 10.1038/s41594-023-00981-1 (PMC10191856; doi:10.1038/s41594-023-00981-1)

205 kDa

120 kDa

85 kDa

65 kDa

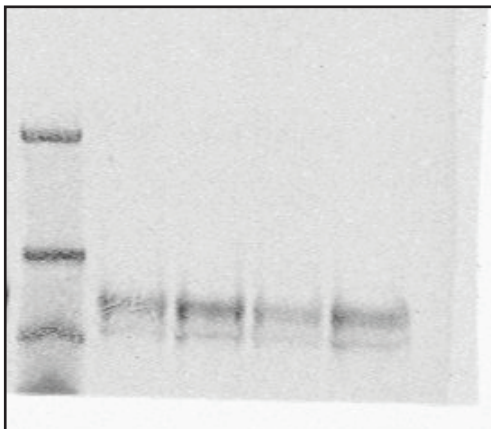

Gal4-3V5

**V5 antibody**

50 kDa

30 kDa

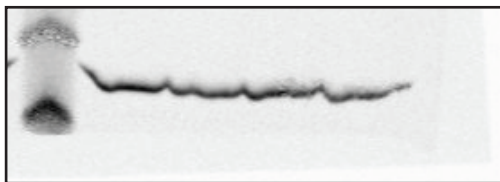

Pgk1

**Pgk1 antibody**

Supplement: Source Data Extended Data Fig. 3 — Unprocessed western blots. [file 41594_2023_981_MOESM14_ESM.pdf]

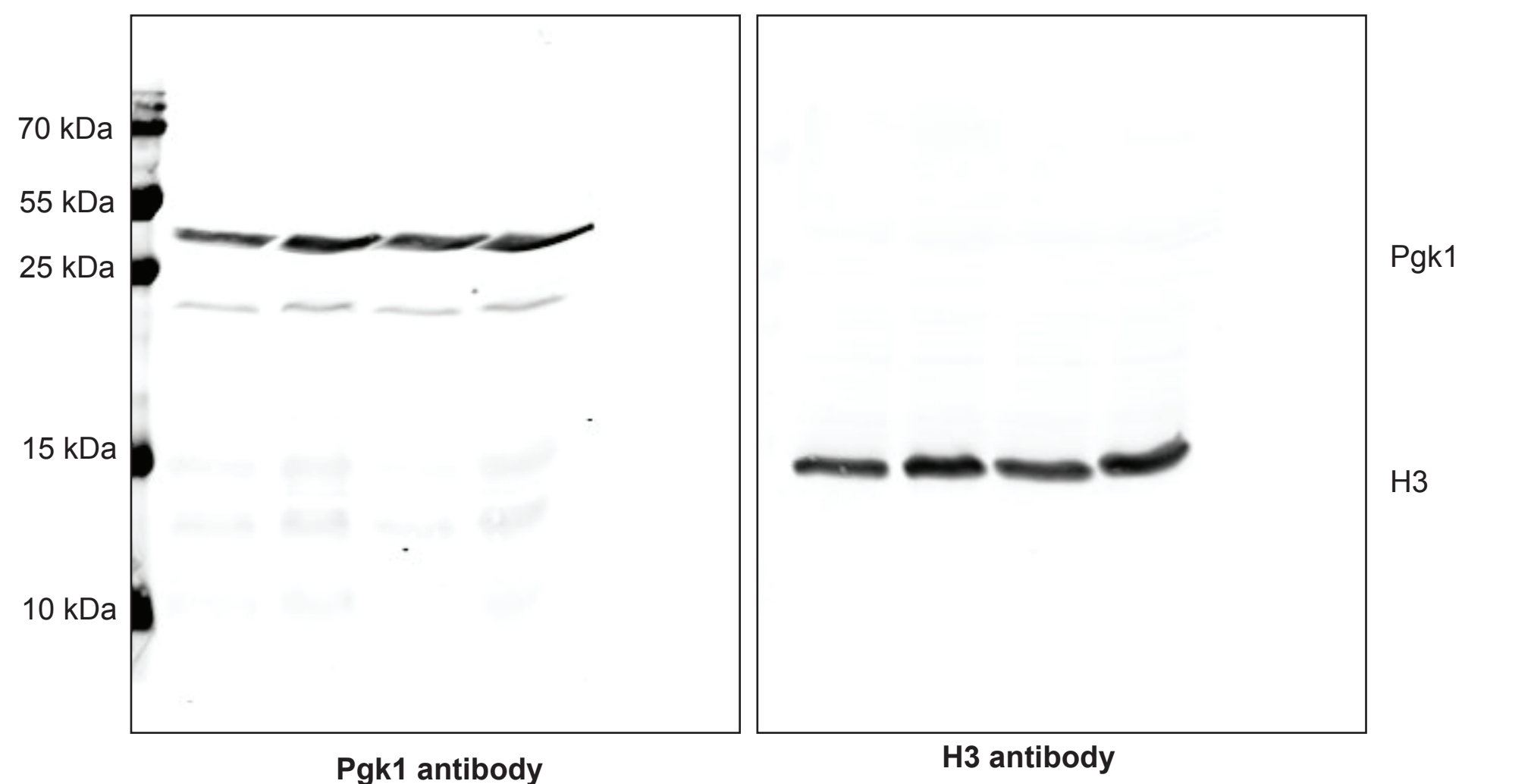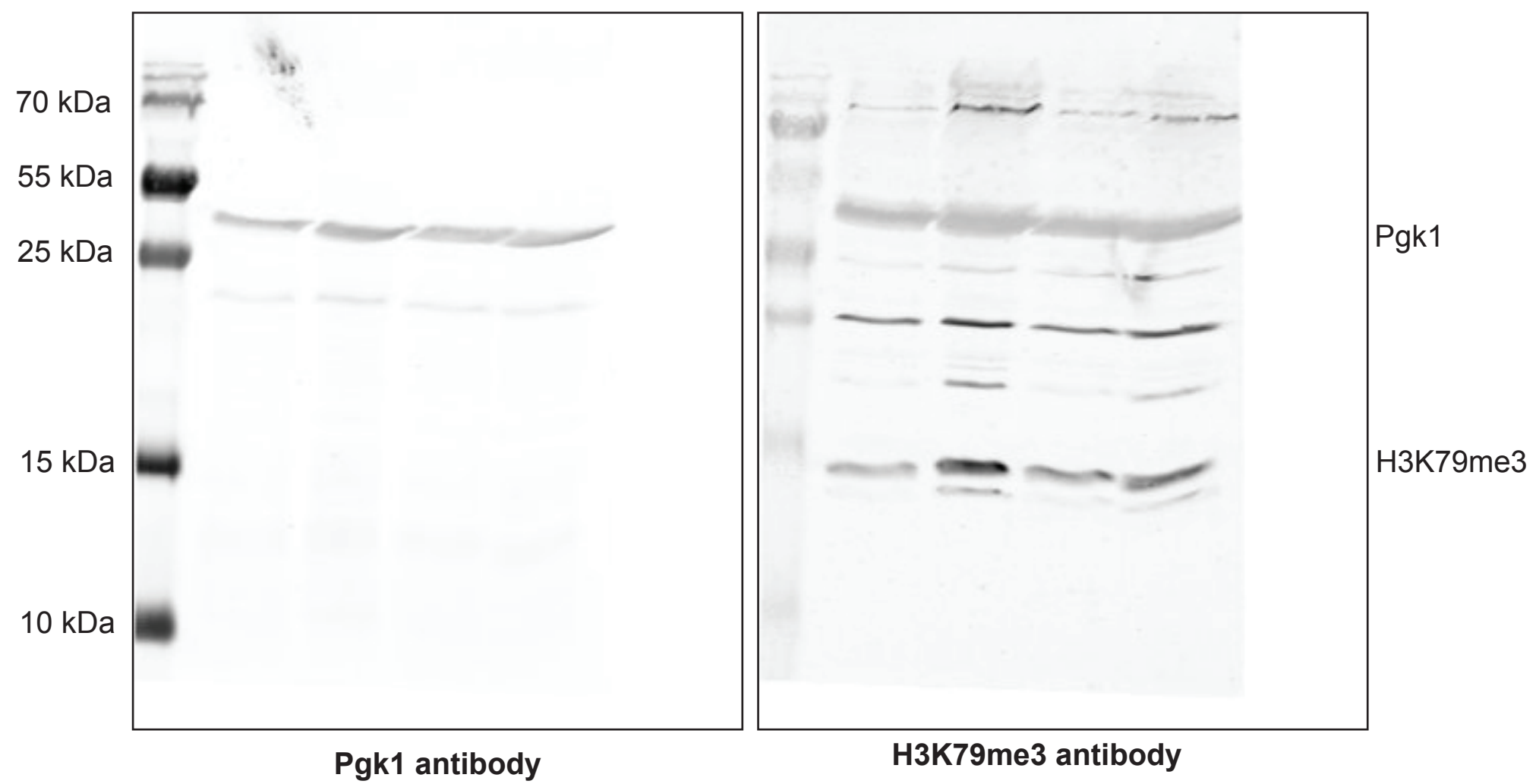

Supplement: Source Data Extended Data Fig. 8 — Unprocessed western blots. [file 41594_2023_981_MOESM20_ESM.pdf]
